# Recombinant Human Superoxide Dismutase and *N*-Acetylcysteine Addition to Exogenous Surfactant in the Treatment of Meconium Aspiration Syndrome

**DOI:** 10.3390/molecules24050905

**Published:** 2019-03-05

**Authors:** Jana Kopincova, Maros Kolomaznik, Pavol Mikolka, Petra Kosutova, Juliana Topercerova, Katarina Matasova, Andrea Calkovska, Daniela Mokra

**Affiliations:** 1 Department of Physiology, Jessenius Faculty of Medicine in Martin, Comenius University in Bratislava, Mala Hora 4C, 03601 Martin, Slovakia; kopincova@jfmed.uniba.sk (J.K.); j.topercerova@gmail.com (J.T.); matasova.katka@gmail.com (K.M.J.); calkovska@jfmed.uniba.sk (A.C.); mokra@jfmed.uniba.sk (D.M.); 2 Biomedical Center Martin, Jessenius Faculty of Medicine in Martin, Comenius University in Bratislava, Mala Hora 4C, 03601 Martin, Slovakia; pavol.mikolka@jfmed.uniba.sk (P.M.); petra.kosutova@jfmed.uniba.sk (P.K.)

**Keywords:** meconium aspiration syndrome, surfactant treatment, oxidative stress, superoxide dismutase, *N*-acetylcysteine

## Abstract

This study aimed to evaluate the molecular background of *N*-acetylcysteine (NAC) and recombinant human superoxide dismutase (rhSOD) antioxidant action when combined with exogenous surfactant in the treatment of meconium aspiration syndrome (MAS), considering redox signalling a principal part of cell response to meconium. Young New Zealand rabbits were instilled with meconium suspension (Mec) and treated by surfactant alone (Surf) or surfactant in combination with i.v. NAC (Surf + NAC) or i.t. rhSOD (Surf + SOD), and oxygen-ventilated for 5 h. Dynamic lung-thorax compliance, mean airway pressure, PaO_2_/FiO_2_ and ventilation efficiency index were evaluated every hour; post mortem, inflammatory and oxidative markers (advanced oxidation protein products, total antioxidant capacity, hydroxynonenal (HNE), p38 mitogen activated protein kinase, caspase 3, thromboxane, endothelin-1 and secretory phospholipase A_2_) were assessed in pulmonary tissue homogenates. rhSOD addition to surfactant improved significantly, but transiently, gas exchange and reduced levels of inflammatory and oxidative molecules with higher impact; Surf + NAC had stronger effect only on HNE formation, and duration of treatment efficacy in respiratory parameters. In both antioxidants, it seems that targeting reactive oxygen species may be strong supporting factor in surfactant treatment of MAS due to redox sensitivity of many intracellular pathways triggered by meconium.

## 1. Introduction

Meconium aspiration syndrome (MAS) is a serious respiratory failure of newborns caused by aspiration of meconium-stained amniotic fluid (MSAF) before or during delivery, recently assigned to neonatal acute respiratory distress syndrome (ARDS) [1]. Meconium is the first stool of newborns, and being composed of amniotic fluid, epithelial cells, lanugo, mucus, blood and gastrointestinal secretions, its aspiration leads to mechanical obstruction of airways, surfactant inactivation and initiation of inflammatory pathways [2,3]. Great part of inflammation induced by meconium is mediated by overproduction of free radicals which directly damage biomolecules and induce many intracellular cascades with inflammatory and apoptotic consequences [4].

Medical intervention to oxidant/antioxidant balance had been tried in numerous pulmonary diseases for many years [5,6,7,8,9]. However, as it was pointed out by Greenwald in a critical review, antioxidant therapy makes true sense only in those situations “where oxygen radicals can be truly expected to play a role based on what we know about their biochemistry and pathology” [10]. MAS is on the one hand related to reactive oxygen species (ROS) induction in alveolar macrophages and leukocytes attracted from systemic perfusion due to inflammatory signalling [11,12,13]. On the other hand, in serious MAS, high fractions of oxygen are often needed to maintain sufficient oxygenation of newborns during ventilatory support, which also leads to ROS overproduction, so direct oxidative impairment to biomolecules together with ROS-mediated signalling pathways play important roles in meconium-induced lung injury and injury of the other organs [12,14], and antioxidant therapy in MAS is worthwhile.

When considering antioxidant therapy, two questions are to be addressed: the agent to be used and its delivery route. Generally, antioxidants can be classified as non-enzymatic antioxidants (small molecules, e.g., *N*-acetylcysteine; NAC) and antioxidant enzymes (e.g., superoxide dismutase, SOD) [15]. NAC is a thiol, key precursor of cellular glutathione (GSH) synthesis, which easily penetrates cell membranes and is considered to be “virtually non-toxic” [16]. Aside from effective replenishment of GSH-glutathione peroxidase system converting H_2_O_2_ to H_2_O and O_2_, its sulfhydryl group allows NAC to potently scavenge •OH and HOCl, and poorly O_2_^−^ and H_2_O_2_ [15,17]. In the treatment of respiratory diseases, intravenous (i.v.) or oral NAC had been beneficial in patients with acute lung injury (ALI)/ARDS [5,6]. In our previous experiments, a combination of NAC and surfactant was able to improve gas exchange after meconium aspiration better than the surfactant alone [18], however, at that time we did not reflect too much on the molecular background of NAC-mediated effects on biologically active substances.

Recombinant human SOD (rhSOD) is an antioxidant enzyme eliminating O_2_^−^ by its conversion to H_2_O_2_ which prevents formation of strong oxidants (peroxynitrite and •OH). SOD is naturally present in mammals in three forms, two intracellular and one extracellular. Due to its relatively high molecular weight, rhSOD does not penetrate cellular membranes easily, which hampers its administration [15]. On the other site, its capability to scavenge O_2_^−^ encourages its use in human treatment. rhSOD showed no direct toxicity and had been administered intratracheally (i.t.) to premature infants in doses from 2.5 to 5 mg/kg without side effects [19,20,21].

In MAS, surfactant layer and pulmonary epithelial cells are the first affected by meconium, with subsequent change in the pulmonary vascular tone. However, delivery of antioxidant agents to pulmonary epithelium and endothelium is quite complicated issue, especially for enzymes [15]. Intravascular drug delivery offers homogenous distribution and better access to endothelium. On the other hand, systemic administration of antioxidant enzymes is hindered by their rapid elimination from circulation. “Mega doses” were required to protect the lung, as the fraction found in the lungs does not exceed 1% of the injected dose [15,22]. Intratracheal route of administration, by contrast, facilitates local delivery to airway space; yet large proteins are thought not to cross alveolar-capillary barrier implying lower reachability of pulmonary endothelium; and there is also a possibility of shortened drug lifespan in alveoli [22].

**Scheme 1:**
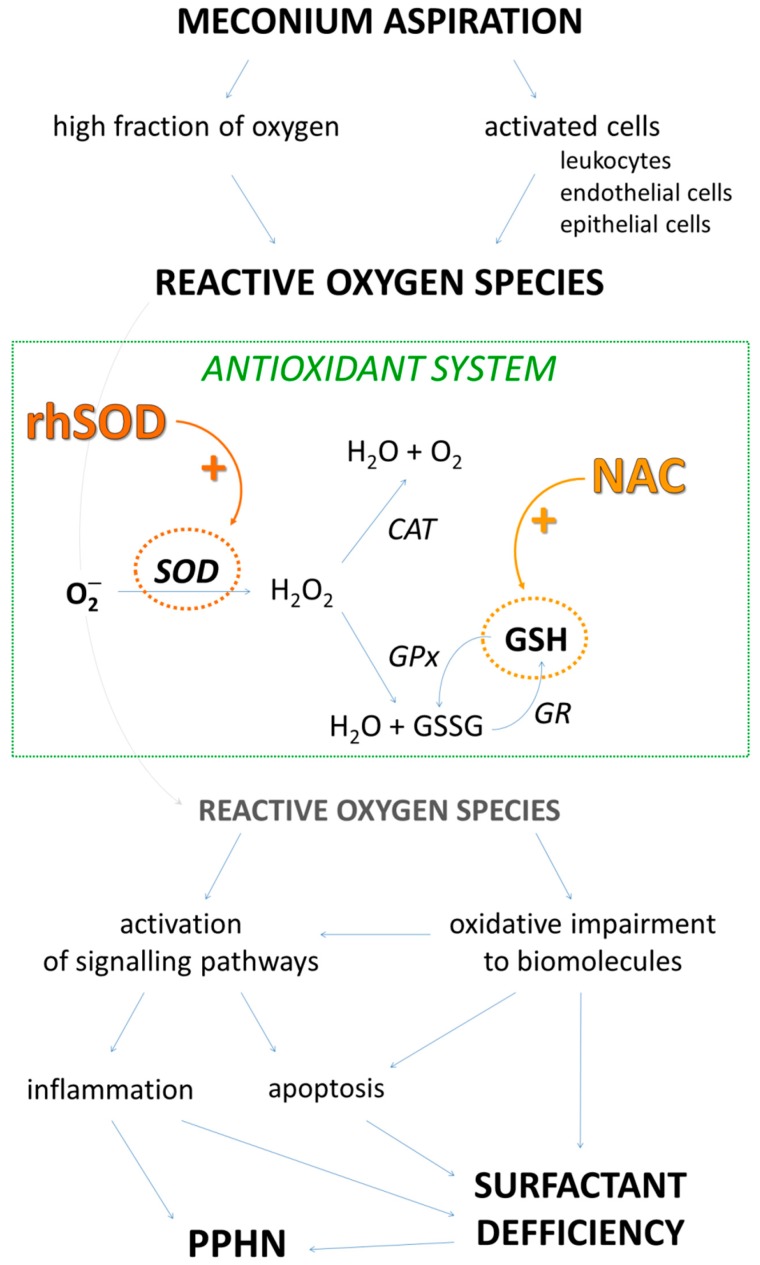
Brief insight into mechanisms induced by reactive oxygen species (ROS) after meconium aspiration. High fraction of oxygen during ventilation and meconium-activated cells contribute to ROS production. Antioxidant system eliminates harmful radicals. Excessive ROS impair biomolecules and trigger pathways leading to inflammatory and apoptotic processes. As a result, surfactant dysfunction and persistent pulmonary hypertension of newborn (PPHN) can be seen. Administration of either recombinant human superoxide dismutase (rhSOD) or *N*-acetylcysteine (NAC) could increase antioxidant capacity and affect subsequent alterations. SOD—superoxide dismutase; CAT—catalase, GPx—glutathione peroxidase, GR—glutathione reductase, GSH—reduced glutathione; GSSG—oxidized glutathione.

We recently observed high impact of rhSOD addition to exogenous surfactant on respiratory parameters in experimental MAS [23]. However, when we compared the effect of rhSOD combined with surfactant with NAC combined with surfactant from the previous experiments, we found some differences in these two antioxidants. This prompted us to go deeper into the mechanisms and molecular background, which were not analysed in previous NAC studies. In contrast to rhSOD which requires topical administration, NAC might be administered rather systematically. NAC is approved drug, while rhSOD treatment remains experimental to date. Last but not least, each of these antioxidants targets different group of ROS and they are thought to have different antioxidant capacities [15] which might diversely affect serious oxidative impairment in MAS (Scheme 1).

Thus, in this study, we aimed to compare the antioxidant effect of i.t. rhSOD and i.v. NAC combined with surfactant in light of biochemical markers of oxidative stress, apoptosis and inflammatory tissue damage together with respiratory functions in the meconium-instilled rabbit model, which is used as a model for newborns’ MAS [24,25,26]. We suppose that antioxidant enrichment of surfactant treatment may bring additional therapeutic benefit which may somehow differ with different antioxidants.

## 2. Results

### 2.1. Baseline Data

**Figure 1:**
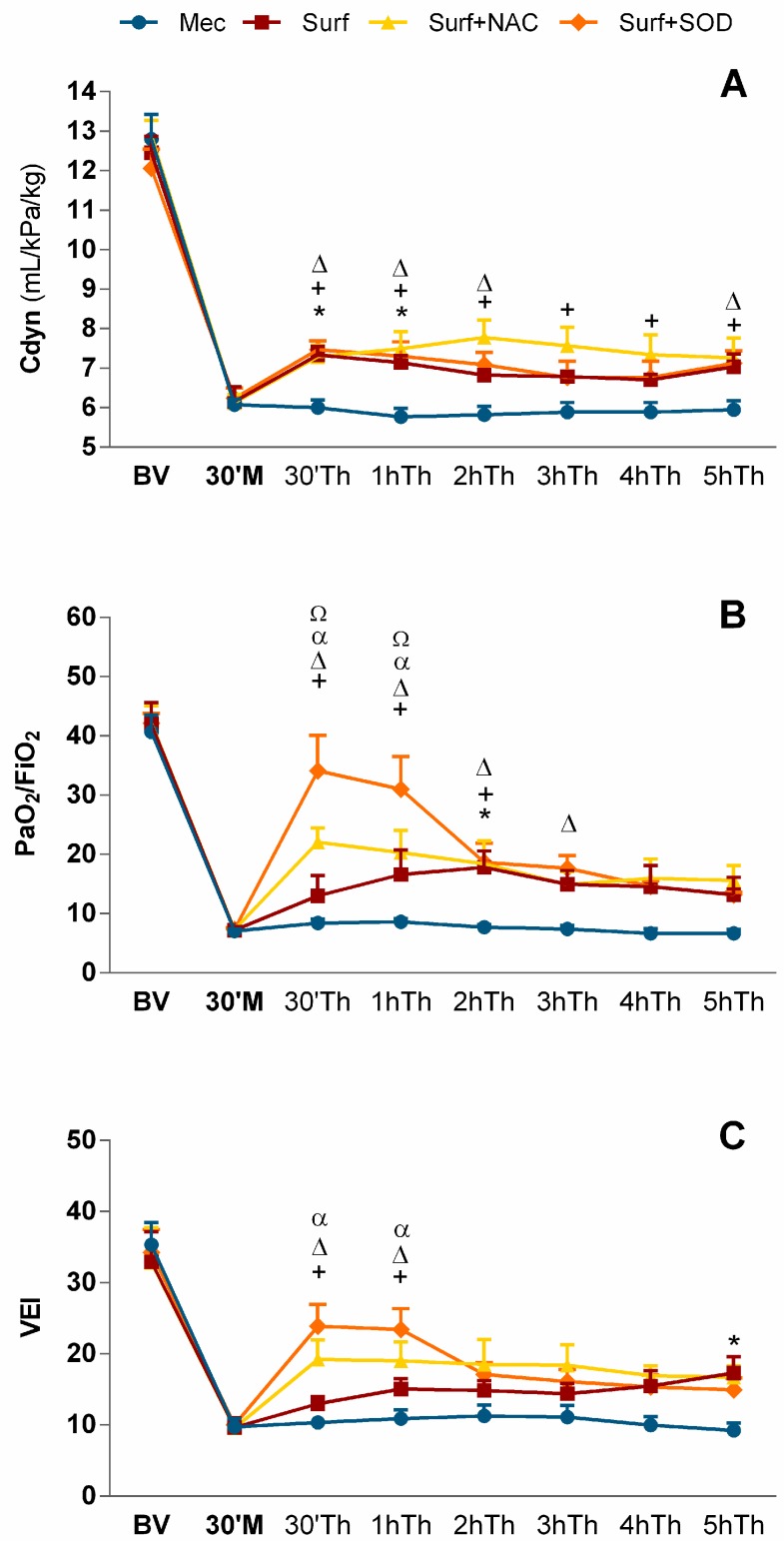
(**A**) Lung compliance (Cdyn), (**B**) PaO_2_/FiO_2_ and (**C**) Ventilation efficiency index (VEI) before meconium administration (BV), 30 min (30′) after meconium (M) and therapy (Th) administration during 5 h (h) of experiment. Mec—untreated meconium group; Surf—surfactant-only treated group; Surf + NAC—surfactant and NAC-treated group; Surf + SOD—surfactant and SOD-treated group; * *p* = 0.041 to 0.018 for Surf vs. Mec; + *p* = 0.038 to 0.0004 for Surf + NAC vs. Mec; ^Δ^ *p* = 0.041 to 0.0001 for Surf + SOD vs. Mec; ^α^ *p* = 0.027 to 0.0001 for Surf + SOD vs. Surf; ^Ω^ *p* = 0.007 to 0.002 for Surf + SOD vs. Surf + NAC. Part of the results reproduced with permission from [23].

Four groups of seven animals were used in this experiment: group instilled with meconium without any treatment (Mec group); group instilled with meconium with surfactant-only treatment (Surf group); group instilled with meconium with combined surfactant and NAC treatment (Surf + NAC group) and group instilled with meconium with combined surfactant and rhSOD treatment (Surf + SOD group). The groups did not differ in body weight (2.41 ± 0.05 kg for Mec, 2.28 ± 0.03 kg for Surf, 2.30 ± 0.06 for Surf + NAC and 2.26 ± 0.07 for Surf + SOD, all *p* > 0.295) nor respiratory parameters before meconium instillation (Figure 1).

### 2.2. Lung Function Parameters

Administration of meconium caused significant worsening in all observed lung function parameters: dynamic lung-thorax compliance (Cdyn), mean airway pressure (MAP), ratio between partial pressure of oxygen in arterial blood to fraction of inspired oxygen (PaO_2_/FiO_2_) and ventilation efficiency index (VEI). Worsening was highly significant when compared to basal values (BV; *p* < 0.0001 vs. BV for each parameter respectively, in all groups), but comparable among groups.

#### 2.2.1. Lung Compliance

Initial values of lung compliance (12.1–12.9 mL/kPa/kg) fell after meconium instillation down to around 6 mL/kPa/kg (*p* < 0.0001 vs. BV) and without treatment, Cdyn remained decreased throughout whole the experiment. Surfactant administration restored values during the first 30 min (30′; *p* = 0.021 in 30′ and *p* = 0.018 in the 1 h vs. Mec), however, the amelioration then slightly faded and oscillated on the border of significance (*p* = 0.086 in 2 h and 0.059 in 5 h of the treatment vs. Mec).

Very similar was the effect of surfactant and rhSOD combination, with temporary improvement in the first 2 h (*p* = 0.010 in 30′, 0.007 in 1 h and 0.027 in 2 h of treatment vs. Mec), low significance in 3h and 4h and recovery in 5 h of the treatment (*p* = 0.041 vs Mec). By contrast, the improvement seen after combination of surfactant and NAC was persistent whole the time with significance varying between *p* = 0.025 to 0.0004 vs. Mec (Figure 1A).

#### 2.2.2. PaO_2_/FiO_2_

PaO_2_/FiO_2_ dropped after meconium instillation from values around 40–42 down to 7.1–7.3 (*p* < 0.0001 vs. BV). Surfactant administration led to transient increase up to 17.8 in 2 h of therapy (*p* = 0.029 vs. Mec). Addition of NAC to the treatment caused rapid rise in PaO_2_/FiO_2_ values to above 22 in 30′ after the treatment (*p* = 0.002 vs. Mec) and for 2 h, oxygenation remained increased (*p* = 0.010 for 1 h and 0.021 for 2 h of treatment vs. Mec). Through the rest of experiment, improvement after Surf + NAC oscillated on the border of significance with *p* = 0.051 in 4 h and 0.060 in 5 h of treatment vs. Mec. Huge impact on PaO_2_/FiO_2_ values was, however, seen in Surf + SOD group; in the first 30′ there was increase up to 34, which was statistically very close to the BV (*p* = 0.061 vs. BV) and amelioration persisted for next 3 h (*p* < 0.0001 in 30′ and 1 h, *p* = 0.018 in 2 h and 0.027 in 3 h of treatment vs. Mec). Moreover, the values were higher compared to surfactant-only treatment (*p* < 0.0001 and *p* = 0.001 in 30′ and 1 h vs. Surf, respectively) and also Surf + NAC treatment (*p* = 0.002 and 0.007 in 30′ and 1 h vs. Surf + NAC, respectively; Figure 1B).

#### 2.2.3. Ventilation Efficiency Index

The ability of the body to eliminate CO_2_ after meconium instillation was strongly restricted (*p* < 0.0001 vs. BV) and without treatment, there was no improvement throughout the time. Surfactant supplementation gradually improved VEI values with significance seen in 5 h (0.041 vs. Mec). Addition of both the antioxidants caused significant amelioration in first 30′ and 1 h (*p* = 0.023 and 0.038 in 30′ and 1 h, respectively for Surf + NAC vs. Mec and *p* = 0.0002 and 0.001 in 30′ and 1 h, respectively for Surf + SOD vs. Mec). Rapid effect of Surf + SOD was higher also compared to Surfactant alone (*p* = 0.004 and 0.027 in 30′ and 1 h, respectively vs. Surf), however, it subsided after 1h of the treatment (non-significant vs. Mec group); whereas impact of Surf + NAC lasted with borderline significance whole the experiment (*p* = 0.064 in 2 h, 0.061 in 3 h, 0.078 in 4 h and 0.058 in 5 h vs. Mec; Figure 1C).

#### 2.2.4. Mean Airway Pressure

Compared to Mec group, in which values of MAP rose from 0.3 kPa to 1 kPa (*p* < 0.0001 vs. BV), all treated groups required lower MAP (*p* < 0.005 for Surf, *p* < 0.0001 for Surf + NAC, *p* < 0.035 for Surf + SOD vs. Mec for whole the experiment). Remarkable improvement was seen in Surf + NAC group in 2 h and 3 h after the treatment, when MAP requirements were significantly lower compared to Surf + SOD group (*p* = 0.016 in 2 h and 0.013 in 3 h of treatment vs. Surf + SOD; Table 1).

### 2.3. Biochemical Parameters

#### 2.3.1. Parameters of Oxidation

**Figure 2:**
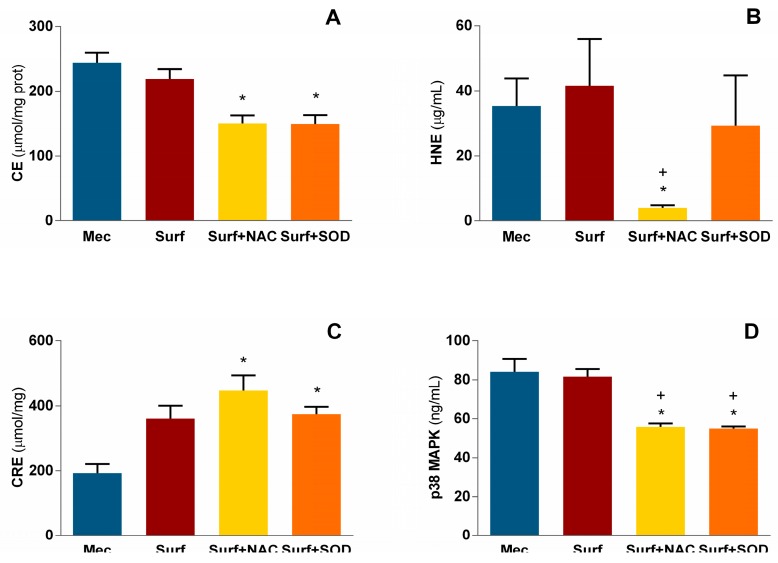
Levels of (**A**) advanced oxidation protein products, (**B**) hydroxynonenal (HNE), (**C**) total antioxidant capacity and (**D**) p38 mitogen-activated protein kinase (p38 MAPK) in pulmonary tissue homogenates. Mec—untreated meconium group; Surf—surfactant-only treated group; Surf + NAC—surfactant and NAC-treated group; Surf + SOD—surfactant and SOD-treated group; CE—chloramine equivalents; CRE—copper reducing equivalents; * *p* = 0.001 to 0.042 vs. Mec; + *p* = 0.015 to 0.046 vs. Surf.

Advanced oxidation protein products had been observed in Mec group in concentration of 244 μmol of chloramine equivalents (CE) and was not affected by surfactant alone, but restricted by addition of both antioxidant substances to about 150 μmol CE (*p* = 0.028 for Surf + NAC vs. Mec and *p* = 0.038 for Surf + SOD vs. Mec; Figure 2A).

Peroxidation of lipids was assessed from hydroxynonenal (HNE) formation. Meconium-treated group value (approximately 35 μg/mL) was markedly suppressed to 4 μg/mL after NAC addition to surfactant treatment with significance compared to Mec group (*p* = 0.025) and also to surfactant alone (*p* = 0.044; Figure 2B), with no effect seen in Surf + SOD group.

Antioxidant capacity of pulmonary tissue was borderline increased in surfactant-treated group (*p* = 0.061 vs. Mec) and increased in Surf + NAC (*p* = 0.002 vs. Mec) and Surf + SOD (*p* = 0.046 vs. Mec) group (Figure 2C).

#### 2.3.2. Parameters of Apoptosis

Meconium instillation evoked in the pulmonary tissue p38 mitogen-activated protein kinase (MAPK) accumulation in amount 84.1 ± 5.6 ng/mL. Both antioxidant supplements limited its concentration similarly, to 55.9 ± 0.7 ng/mL in Surf + NAC group (*p* = 0.013 vs. Mec and 0.018 vs. Surf group), and to 55.0 ± 1.6 ng/mL in Surf + SOD group (*p* = 0.004 vs. Mec and 0.006 vs. Surf group; Figure 2D).

**Figure 3:**
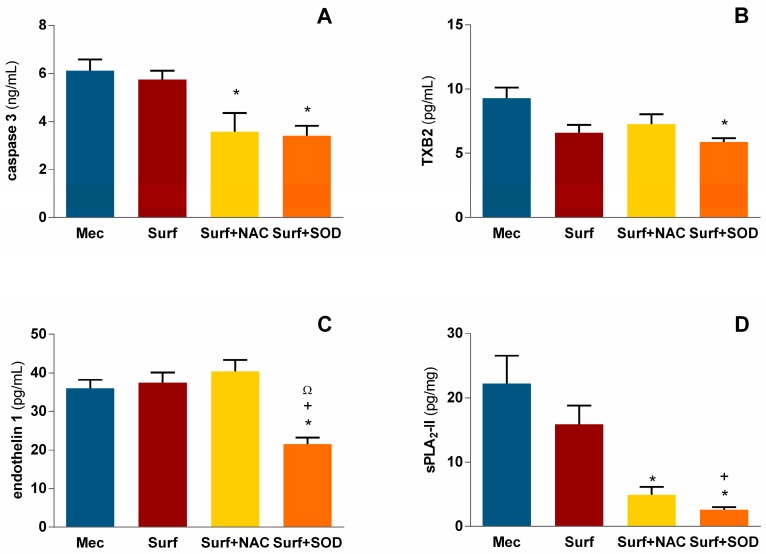
Levels of (**A**) caspase 3, (**B**) thromboxane B2 (TXB2), (**C**) endothelin 1 and (**D**) secretory phospholipase A_2_ type II (sPLA_2_-II) in pulmonary tissue homogenates; Mec—untreated meconium group; Surf—surfactant-only treated group; Surf + NAC—surfactant and NAC-treated group; Surf + SOD—surfactant and SOD-treated group; * *p* = 0.009 to 0.046 vs. Mec; + *p* = 0.011 to 0.013 vs. Surf; ^Ω^ *p* = 0.002 vs. Surf + NAC.

In case of caspase 3, both Surf + NAC and Surf + SOD treatment restricted its formation from Mec and Surf values between 5.7–6.1 ng/mL down to 3.4–3.6 ng/mL (*p* = 0.028 for Surf + NAC and *p* = 0.014 for Surf + SOD vs. Mec; Figure 3A).

#### 2.3.3. Biologically Active Substances

Vasoactive thromboxane A2 (TXA2) has half-life around 30 s; by virtue of its rapid degradation in vivo, TXA2 levels are often inquired by assessing its more stable metabolite thromboxane B2 (TXB2) [27]. In our experiment, meconium administration caused TXB2 production on level 9.3 ± 0.8 pg/mL and it was borderline dampened by surfactant therapy down to 6.6 ± 0.6 pg/mL (*p* = 0.089 vs. Mec) and dampened by Surf + SOD therapy to 5.9 ± 0.3 pg/mL (*p* = 0.009 vs. Mec; Figure 3B).

Endothelin-1 (ET-1) concentration in pulmonary tissue was similar in Mec, Surf and Surf + NAC groups and reached values more than 36 pg/mL. Addition of rhSOD diminished ET-1 production down to 21.5 ± 1.7 pg/mL, which was significantly lower compared to all three forenamed groups (*p* = 0.046 vs. Mec, 0.013 vs. Surf and 0.002 vs. Surf + NAC group; Figure 3C).

Meconium is known to both contain and induce secretory phospholipase A_2_ (sPLA_2_); the type II, or pulmonary sPLA_2_, which was examined in this study reached values about 22 pg/mg in untreated group, and was not affected by surfactant alone; however, addition of NAC to surfactant treatment led to decrease of sPLA_2_ down to 4.9 ± 1.2 pg/mg (*p* = 0.025 vs. Mec), and addition of SOD caused decrease to 2.6 ± 0.4 pg/mg with significance against both Mec group (*p* = 0.001) and Surf group (*p* = 0.011; Figure 3D).

## 3. Discussion

Meconium aspiration has deleterious consequences on the airways and lungs of newborn. Airway obstruction by this viscous substance is further worsened by surfactant inactivation, triggering of inflammatory pathways and often complicated by persistent pulmonary hypertension (PPHN) [28]. A great part of inflammatory processes results from the presence of abnormal amounts of reactive oxygen species affecting redox-sensitive intracellular signalling, and directly injuring structural and functional biomolecules of the tissue [11]. The source of oxidative species can be found in leukocytes, endothelial and epithelial cells which are activated by cytokines and harmful compounds contained in meconium [29,30], and by compounds produced in pulmonary tissue in response to meconium presence [4]. The other source of ROS originates in higher fractions of oxygen in the inhaled air which is often needed to keep oxygenation through obstructed lungs [31]. As a consequence of high oxidant accumulation, surfactant impairment, alveolar-capillary membrane damage with protein leakage, induced cellular necrosis and apoptosis and production/activation of unwanted substances can be seen [3,31].

Bronchoalveolar lavage with diluted surfactant helps to at least partially remove meconium and locally produced pro-inflammatory mediators [31]. The inactivated endogenous surfactant might then be restored by intratracheal surfactant bolus; however, despite some observed benefits in pulmonary oxygenation, surfactant treatment in MAS is still discussed due to lack of effect on the other outcomes [28]. In our opinion, unsatisfactory efficacy of surfactant treatment could be caused by its later inactivation by persistent inflammation in the presence of residual meconium. In inflamed lungs, activated cells produce huge quantities of ROS which first of all attack surfactant phospholipids and then also surrounding tissue, resulting in release of neutrophil-derived proteases and secretory phospholipases A_2_ detrimental for surfactant molecules [3,9,29].

In our experiments, surfactant treatment lowered meconium-increased lung compliance for 1 h and then oscillated on the border of significance. rhSOD addition managed to improve the effect of surfactant only partially—the initial impact lasted an h longer and then became visible again at the 5th h of the treatment. In contrast, systemic administration of NAC was able to sustain improvement throughout the whole time of the experiment.

Decrease in lung compliance is associated with surfactant inactivation. rhSOD addition should be able to prevent oxidative damage to surfactant molecules in the very first step [32]; rhSOD is able to scavenge O_2_^−^, the first radical formed during oxygen therapy which gives the basis for harmful peroxynitrite or hydroxyl radical [33]. However, the results suggest that rhSOD was early inactivated, perhaps due to residual meconium and persistent inflammation. There are many factors which might contribute to rhSOD inactivation in the course of time, e.g., accumulated hydrogen peroxide (especially in case when there is not enough catalase capacity to remove it) [15,34], macrophage-derived nitric oxide, peroxynitrite, malondialdehyde, 4-hydroxynonenal or fetal pancreatic proteolytic enzymes contained in meconium [35,36].

The idea of rhSOD inactivation is also supported by rhSOD effect on oxygenation. rhSOD addition led to rapid improvement, highly increased even compared to surfactant alone and also surfactant combined with NAC, but after three h this effect disappeared (values came back to 13–14 and were very same as in surfactant-only treated group). NAC addition elevated PaO_2_/FiO_2_ for the first three h with significance mildly below 0.05, but then the effect remained borderline till the end. Similar was the course of VEI, with magnificent Surf + SOD effect which faded out early, and moderate significant effect of Surf + NAC which lasted oscillating on the border by the end of the experiment. After i.v. administration, terminal half-life of NAC is around 5 h [15,37]; thus even when bioavailability is just around 10% of the dose [15], it seems that the time of its duration was sufficient to bring improvement in Cdyn and gas exchange visible whole our experiment.

All changes in pulmonary status of treated animals were also reflected in ventilation pressures needed to keep sufficient oxygenation. Supplementation of surfactant led to decrease in MAP in all three therapies, however, the effect of NAC addition in the 2 h and 3 h of treatment (the time when it seemed that SOD started to be inactivated) showed to be more effective than SOD addition.

Which mechanisms may be responsible for these actions? It is worth mentioning that besides antioxidant function, NAC has also the ability to cleave disulphide bonds and decrease the tenacity of meconium [38]—this could support mucociliary clearance [39]. The other possible mechanisms are shared by both the added antioxidants.

When we analysed pulmonary tissue post mortem, there was surprising difference between the effect of NAC and SOD addition on HNE production. It was inhibited by NAC, but not changed in SOD presence, while other parameters of oxidant/antioxidant status were affected similarly by both the agents. This result was unexpected and we can just speculate about the reason of rhSOD inefficiency. It is known that lipids are the first target for oxidation, so maybe HNE accumulation was the result of remaining h of experiment after rhSOD instant breakdown. Or maybe, SOD itself contributed to HNE formation, because in the absence of adequate H_2_O_2_ detoxification, SOD may also aggravate oxidative stress [15]. By return, increased HNE could affect rhSOD activity.

Many inflammatory cascades are redox-sensitive, meaning that intracellular reduction and oxidation changes are involved in the activation of their signal molecules and pathways. In our previous works, we speculated about meconium-associated p38 MAPK activation that is also redox-sensitive [18,23]. p38 MAPK is activated by both the presence of ROS and Toll-like receptor 4 (TLR4) signalling pathway—the one used by meconium [40]. It had been shown that pre-incubation of human bronchial epithelial cells with NAC attenuates tumor necrosis factor α (TNF-α)-induced p38 MAPK activation via glutathione-dependent mechanism [41]. Consistently with this observation, diminishing of free radicals by both antioxidants restricted p38 MAPK activation similarly when compared to untreated and also surfactant-treated group.

p38 MAPK attracted our attention due to its association with apoptotic caspase 3 and cyclooxygenase 2 (COX-2) activation [41,42]. Meconium is known to initiate processes of necrosis and apoptosis of human epithelial cells in dose- and time-dependent manner, and these processes are caspase-mediated [43]. This may be possibly ascribed to digestive proteolytic enzymes contained in meconium [35] and/or to local angiotensin II (Ang II) production [4] or sPLA_2_ activity [44]. Addition of bovine surfactant was shown to have a tendency to prevent meconium-induced tissue necrosis in piglet model [45]. In our case, caspase 3 was restrained in both antioxidant-treated groups, but not in surfactant-only-treated group, in accordance with Jeng who did not observed caspase inhibition in meconium-stimulated epithelial cells in the presence of exogenous surfactant [43]. Antioxidant-dependent restriction in meconium-induced caspase level may point the role of ROS in this process.

COX-2 is associated with prostanoid synthesis using arachidonic acid as a substrate. It is being expressed after initial stimulus which can be IL-1β, TNF-α, TLR4 ligand, oxidative stress, trypsin or other substances, using several pathways including those of MAPK, extracellular-signal-regulated kinase (ERK) and NF-κB [46]. Prostanoid production takes place in bronchial and alveolar epithelium, in airway smooth muscle cells and vascular endothelium [27,41,46,47]. Some of prostanoids, e.g., prostaglandin D2 and thromboxane A2 are potent bronchoconstrictors [27,46,48] and the latter also vasoconstrictor [28]. Acute perinatal asphyxia or alveolar hypoxia cause pulmonary vessels to constrict and meconium-induced production of thromboxane and endothelin even promotes the development of pulmonary hypertension [49]. In our experiments, thromboxane levels were not tightly correlated with p38 MAPK suppression. TXB2 values were decreased only in Surf + SOD group, with a tendency to decrease also in Surf group; and NAC administration did not affect TXB2. This difference requires further elucidation; it might be caused by distinct, non-redox-sensitive pathway of COX induction, or it might be someway linked with sPLA_2_-II which may be conducive to the production of pro-inflammatory eicosanoids [50] or directly to arachidonic acid release [51,52]. And we cannot exclude the possibility that the main source of TXB2 was in meconium-stimulated epithelial cells [53] and its suppression was caused by the direct and strong antioxidant activity of i.t. given rhSOD.

In addition to COX, pulmonary circulation is controlled also by ET-1 production. Elevated levels of ET-1 in experimental MAS models have been observed both in the plasma and bronchoalveolar lavage fluid [24,54,55] and selective blockage of ETA receptors revealed huge impact of ET-1 on hemodynamic parameters and oxygenation index after meconium instillation [56]. In addition to increase in vascular tone, ET-1 was reported to mediate bronchoconstriction which was even more pronounced in case of airway epithelium injury [57]. The reason for ET-1 overproduction in MAS is not comprehensively answered; there are more factors contributing to pulmonary ET-1 synthesis. First of all, increase in ROS production is thought to trigger different mechanisms of hypoxic pulmonary vasoconstriction. Among them, there is ROS-mediated elevation of hypoxia-inducible factor 1α (HIF-1α), a transcription factor targeting ET-1 gene under hypoxic conditions [58,59]. Additionally, it was found that Ang II increases ET-1 expression in smooth muscle cells via redox-sensitive ERK pathway; the involvement of ROS in this process was confirmed by antioxidant application [60]. Similarly to TXB2, ET-1 is also produced in epithelial cells of respiratory system [57,61], and its production was in our experiment suppressed only after rhSOD addition as well. Thus, it further supports the idea of direct impact of rhSOD on airway epithelium responses.

Meconium is also known to contain sPLA_2_ type IB (pancreatic), and to induce type IIA (pulmonary) sPLA_2_, which was associated with surfactant dysfunction and lung injury [29]. In our experiments, both antioxidants managed to decrease the level of sPLA_2_-IIA in tissue homogenates, with higher grade seen in Surf + SOD treatment. This improvement cannot be ascribed just to meconium lavage, as surfactant treatment alone did not affect sPLA_2_-IIA presence, and rhSOD addition succeeded significantly when compared to surfactant-only treated group, suggesting the role of antioxidant/oxidant balance in sPLA_2_-IIA induction. Considering many harmful effects of sPLA_2_ in meconium-instilled lungs [29], its repression signifies appreciable benefit. Beside arachidonic acid release, some of sPLA_2_s can also activate ERK1/2 pathway and/or p38 MAPK pathway resulting in cytokine, chemokine and inductive NO synthase production [62,63], thus closing the circle of inflammatory signalling and prostaglandin production.

Considering our results, it seems that antioxidant treatment in MAS may suppress some crucial pathways; however, there is still the question of the administration method. The distinct routes of administration for rhSOD and NAC, on the one hand, a limitation of this study. On the other hand, these conditions helped to reveal differences in their therapeutic duration and efficacy. Large antioxidant enzymes such as rhSOD are thought not to cross membranes and the endothelium is almost unavailable for them [15]. Actually, experimental i.t. rhSOD delivery led to enzyme incorporation into pulmonary epithelial cells within one h and caused 100% increase in SOD activity in lungs [64], and in rhSOD-instilled newborns and newborn lambs, serum levels of the enzyme were increased over couple of h [19,65]. Moreover, using exogenous surfactant as a vehicle may further enhance the homogeneity of distribution, protect the enzyme from pulmonary clearance and facilitate SOD uptake by epithelial cells [66,67,68]. Contrary to rhSOD, NAC features high cell membrane permeability and the clinical routes of administration comprise of oral, i.v. and topical (e.g., as aerosol) [69]. Beside a lot of benefit, there are also some detriments observed after topical NAC administration. For example, aerosol delivery may lead in NAC oxidation and dimerization with negative consequences and biphasic effect of the treatment, due to NAC acting as pro-oxidant [70]. Intratracheal NAC increased total airway resistance and bradycardia episodes in pre-term neonates with bronchopulmonary dysplasia [71] and nebulised NAC did not affected respiratory distress syndrome in premature infants [72], suggesting rather i.v. administration.

## 4. Materials and Methods

### 4.1. Animals

The protocol of our experiments (Project identification code VEGA 1/0291/12 and VEGA 1/0356/18) was approved by the local Ethics Committee of Jessenius Faculty of Medicine, Comenius University and National Veterinary Board of Slovak Republic (Ro-2168/11-221 on 23/09/2011 and Ro-4857/17-221 on 22/01/2018). The protocol follows EU Directive 2010/63/EU for animal experiments and comply with the ARRIVE guidelines.

Young New Zealand white rabbits (28) of both genders, with body weights (b.w.) of 2.1 to 2.5 kg and approximately 8–11 weeks of age were obtained from Velaz s. r. o. (Praha, Czech Republic). They were housed one or two per cage in transparent cages with a plastic bottom grid without bedding, provided by an elevated resting platform and enhanced by wooden blocks, at a temperature of 20 °C and 60% humidity, and under 12/12 h light/dark cycle. Rabbits were fed standard diet (Velaz s. r. o.) once per day according to the weight range and the access to water was ad libitum.

### 4.2. Procedure

#### 4.2.1. Experimental Design

Around 8 AM, each animal underwent anesthetic and surgical procedures described earlier [18,73]—in brief, intramuscular tiletamine + zolazepam (15 mg/kg b.w.; Zoletil, Virbac, France) and xylazine (5 mg/kg b.w.; Xylariem, Riemser, Germany), followed by infusion of tiletamine + zolazepam (10 mg/kg/h i.v.), were administered; tracheotomy and catheterisation of the femoral artery and right atrium for blood sampling and blood pressure measuring, and of the femoral vein for drug and anesthetics administration was done; paralysis was made by pipecuronium bromide (0.3 mg/kg b.w./30 min; Arduan, Gedeon Richter, Hungary); then the animal was left to stabilize for 15 min on conventional IPPV (intermittent positive pressure ventilation) mode (Beat-2, Chirana, Stará Turá, Slovakia) set on frequency of 30/min, fraction of inspired oxygen (FiO_2_) of 0.21, inspiration time Ti 50%, peak inspiratory pressure (PIP) set to keep tidal volume (VT) between 7–9 mL/kg b.w., and no positive end-expiratory pressure (PEEP). When stabilized, lung function parameters were recorded (VT, PIP, PEEP) and blood gases in arterial blood samples were evaluated (PaO_2_, PaCO_2_) using RapidLab 348 (Siemens, Eschborn, Germany) to get basal values (BV).

#### 4.2.2. Experimental Groups

The animals were assigned into four groups: (1) meconium administration without any treatment (Mec group, *n* = 7); (2) meconium administration followed by surfactant-only treatment (Surf group, *n* = 7); (3) meconium administration followed by combined surfactant and NAC treatment (Surf + NAC group, *n* = 7); (4) meconium administration followed by combined surfactant and rhSOD treatment (Surf + SOD group, *n* = 7). Each experimental day the group assignment was done and the choice of the animal to be examined was done by the staff of faculty animal house that was not informed about the type of experiments or the treatment groups. The animal experiments were done in two longer periods, separated by the pause of few months. Results of respiratory parameters of groups Mec, Surf and Surf + SOD were already published and are used with permission from [23].

#### 4.2.3. Meconium Instillation

All the rabbits were administered two equal portions of a dose of 4 mL/kg b.w. of meconium suspension (25 mg/mL), while being positioned to the right and left. Then PEEP was set to 0.25–0.3 kPa and FiO_2_ increased to 1.0. The model of MAS was established within 30 min of meconium instillation, when dynamic lung-thorax compliance (Cdyn) decreased for more than 30% and PaO_2_ < 10 kPa, indicating respiratory failure. At this point, lung function parameters and blood gasses were recorded again.

#### 4.2.4. Treatment Protocol

All surfactant-treated groups received modified porcine surfactant, poractant alfa (Curosurf^®^, Chiesi Farmaceutici, Parma, Italy; 80 mg phospholipids (PL)/mL) in two-phase process consisting of two lavages with diluted Curosurf^®^ (PL concentration of 5 mg/mL and volume of 10 mL/kg b.w.) and undiluted Curosurf^®^ bolus at a dose of 100 mg PL/kg, 1.25 mL/kg b.w. (for details see [18,73]). In Surf + NAC-treated group, i.v. *N*-acetylcysteine (10 mg/kg b.w.; ACC Injekt, Salutas Pharma GmbH, Barleben, Germany) was given right after bolus surfactant dose. In Surf + SOD group, 5 mg/kg b.w. of rhSOD (recombinant human copper, zinc-superoxide dismutase, LifeTein LLC, Somerset, NJ, USA) was dissolved in 0.5 mL of saline and added to surfactant bolus. All animals (treated and not-treated) were oxygen-ventilated using IPPV (FiO_2_ 1.0, frequency 30/min, VT 7–9 mL/kg b.w.) for following 5 h. At time of 30 min, 1, 2, 3, 4, and 5 h after the treatment or in the equivalent time, lung function parameters and blood gases were recorded. Animals were sacrificed by an overdose of anesthetics at the end of experiment.

### 4.3. Measurement and Calculation of Lung Functions Parameters

During ventilation, tracheal airflow had been measured by heated Fleisch head connected to pneumotachograph (UMMT SAV, Bratislava, Slovakia). There was pneumatic catheter placed in the tracheal tube and connected to electromanometer for registration of airway pressure (Tesla, Valašské Meziříčí, Czech Republic). Cdyn was calculated as ratio between the tidal volume per kg b.w. and the airway pressure gradient (PIP-PEEP). Mean airway pressure (MAP) was calculated as MAP = (PIP + PEEP)/2; ventilation efficiency index (VEI) as VEI = 3800/[(PIP − PEEP)] × frequency × PaCO_2_.

### 4.4. Biochemical Analyses in Lung Tissue Homogenates

#### 4.4.1. Homogenisation

Pulmonary tissue strips were homogenized in ice-cold PBS (0.02 mol/L, pH 7.2) to final weight/volume concentration of 10%. Homogenates underwent two freeze-thaw cycles and then were centrifuged at 5000× *g*/5 min at 4 °C. Supernatants were removed and analysed. The examiners of biochemical analyses were blinded to group allocation of investigated animals.

#### 4.4.2. Assessment of Oxidative Stress

Protein oxidation was measured using OxiSelect™ AOPP Assay Kit (Cell Biolabs Inc., San Diego, CA, USA) for advanced oxidation protein products (expressed as μmol of chloramine equivalents (CE)/mg of tissue protein). Lipid peroxidation had been evaluated by OxiSelect™ HNE Adduct Competitive ELISA Kit (Cell Biolabs Inc.) for hydroxynonenal (HNE) protein adducts (expressed as μg/mL of tissue homogenate). Total antioxidant capacity was assessed by OxiSelect™ Total Antioxidant Capacity (TAC) Assay Kit (Cell Biolabs Inc.) expressed as μmol of copper reducing equivalents (CRE)/mg of tissue.

#### 4.4.3. Assessment of Apoptosis Markers

Markers of apoptosis (p38 mitogen-activated protein kinase (p38 MAPK) and caspase 3) were measured by p38 MAPK alpha ELISA Kit (Abcam, Cambridge, UK) and Rabbit Caspase 3 ELISA Kit (Cusabio, Wuhan, China) according to the manufacturer’s instructions. Data were expressed as ng/mL of tissue homogenate.

#### 4.4.4. Assessment of Biologically Active Substances

Thromboxane B2 (TXB2) was measured by Thromboxane B2 ELISA Kit (LifeSpan Biosciences, Seattle, WA, USA). Endothelin 1 was measured by Rabbit Endothelin 1 (ET-1) ELISA Kit (Cusabio); both were expressed as pg/mL of tissue homogenate.

Secretory phospholipase A_2_ (sPLA_2_) type II was analysed using sPLA_2_ (human Type IIA) ELISA Kit (Cayman Chemical Company, Ann Arbor, MI, USA) and expressed as pg/mg of tissue.

### 4.5. Statistical Analyses of Results

Statistical analyses were done by STATISTICA (version 10, StatSoft, Inc., Praha, Czech Republic). For Cdyn, MAP, PaO_2_/FiO_2_, and VEI, Two-way analysis of variance (ANOVA, grouping factors “group” and “time”) with Duncan post-hoc test was used. Non-parametric analysis (Kruskal-Wallis ANOVA test with post-hoc comparisons of mean ranks for all groups) was used for comparison of all biochemical parameters as data did not have normal distribution. A value of *p* < 0.05 was considered to be statistically significant. Numeric values are expressed as mean ± standard error of mean (SEM).

## 5. Conclusions

SOD addition to surfactant treatment of experimental MAS was at first sight very efficient but transient concerning respiratory parameters; however, the invisible molecular effect revealed by post mortem tissue analyses indicates its antioxidant activity. The main disadvantage which remains for other investigations is its short-term effect and the way of administration. The i.v. NAC, in rather low dose, brought milder improvement of respiratory parameters which endured borderline throughout whole the experimental time, however, with a bit less molecular impact than rhSOD. The question remains if the increase in NAC dose would be more powerful (in clinical practice, doses of 150 mg/kg and more are sometimes used [74]) or if some modification (e.g., encapsulation) of the molecule would help [75]. And there is also an option to combine these two agents in MAS treatment. Given NAC ability to distribute adequately throughout pulmonary tissue and eliminate H_2_O_2_, rhSOD activity might not be inhibited as rapidly. Differences in substrates and in permeability of these two agents could be beneficial if used together.

## Figures and Tables

**Table 1:** Mean airway pressure (kPa) before meconium administration (BV) and 30 min (30′) after meconium (Mec) and therapy (Th) administration during 5 h of experiment.

	BV	30′ Mec	30′ Th	1 h	2 h	3 h	4 h	5 h
Mec	0.30 ± 0.01	0.97 ± 0.02	0.98 ± 0.02	0.98 ± 0.01	0.99 ± 0.02	0.98 ± 0.02	0.99 ± 0.02	1.00 ± 0.02
Surf	0.33 ± 0.01	0.93 ± 0.03	0.77 ± 0.02 *	0.79 ± 0.01 *	0.80 ± 0.02 *	0.81 ± 0.02 *	0.86 ± 0.02 *	0.85 ± 0.02 *
Surf + NAC	0.31 ± 0.01	0.95 ± 0.02	0.79 ± 0.04 ^+^	0.76 ± 0.04 ^+^	0.74 ± 0.04 ^+X^	0.76 ± 0.05 ^+X^	0.80 ± 0.04 ^+^	0.82 ± 0.04 ^+^
Surf + SOD	0.35 ± 0.02	0.96 ± 0.02	0.80 ± 0.03 ^Δ^	0.83 ± 0.04 ^Δ^	0.85 ± 0.03 ^Δ^	0.88 ± 0.04 ^Δ^	0.88 ± 0.04 ^Δ^	0.84 ± 0.04 ^Δ^

Mec—untreated meconium group; Surf—surfactant-only treated group; Surf + NAC—surfactant and NAC-treated group; Surf + SOD—surfactant and SOD-treated group; * *p* = 0.005 to 0.0001 for Surf vs. Mec; ^+^ *p* < 0.0001 for Surf + NAC vs. Mec; ^Δ^ *p* = 0.035 to 0.0001 for Surf + SOD vs. Mec; ^X^ *p* = 0.013 to 0.016 for Surf + NAC vs. Surf + SOD.
